# The Characteristics of Long-Wave Irregularities in High-Speed Railway Vertical Curves and Method for Mitigation

**DOI:** 10.3390/s24134403

**Published:** 2024-07-07

**Authors:** Laiwei Jiang, Yangtenglong Li, Yuyuan Zhao, Minyi Cen

**Affiliations:** 1 Faculty of Geosciences and Engineering, Southwest Jiaotong University, Chengdu 611756, China; jianglaiwei@163.com; 2 Key Laboratory of High-Speed Railway Engineering of Ministry of Education, Southwest Jiaotong University, Chengdu 610031, China; liyangtenglong17@cdut.edu.cn; 3 Key Laboratory of Roads and Railway Engineering Safety Control of Ministry of Education, Shijiazhuang Tiedao University, Shijiazhuang 050043, China; 4 College of Earth and Planetary Sciences, Chengdu University of Technology, Chengdu 610059, China; 5 Track Maintenance Department, China Railway Guangzhou Group Co., Ltd., Guangzhou 510088, China; 13926285636@139.com

**Keywords:** track geometry measurements, track geometry cars, high-speed railway, vertical curve, long-wave irregularities, transition curve

## Abstract

Track geometry measurements (TGMs) are a critical methodology for assessing the quality of track regularities and, thus, are essential for ensuring the safety and comfort of high-speed railway (HSR) operations. TGMs also serve as foundational datasets for engineering departments to devise daily maintenance and repair strategies. During routine maintenance, S-shaped long-wave irregularities (SLIs) were found to be present in the vertical direction from track geometry cars (TGCs) at the beginning and end of a vertical curve (VC). In this paper, we conduct a comprehensive analysis and comparison of the characteristics of these SLIs and design a long-wave filter for simulating inertial measurement systems (IMSs). This simulation experiment conclusively demonstrates that SLIs are not attributed to track geometric deformation from the design reference. Instead, imperfections in the longitudinal profile’s design are what cause abrupt changes in the vehicle’s acceleration, resulting in the measurement output of SLIs. Expanding upon this foundation, an additional investigation concerning the quantitative relationship between SLIs and longitudinal profiles is pursued. Finally, a method that involves the addition of a third-degree parabolic transition curve (TDPTC) or a full-wave sinusoidal transition curve (FSTC) is proposed for a smooth transition between the slope and the circular curve, designed to eliminate the abrupt changes in vertical acceleration and to mitigate SLIs. The correctness and effectiveness of this method are validated through filtering simulation experiments. These experiments indicate that the proposed method not only eliminates abrupt changes in vertical acceleration, but also significantly mitigates SLIs.

## 1. Introduction

Track irregularities (TIs) are the primary source of excitation in a track vehicle, constituting a fundamental cause of vehicle vibration and shaking [1,2,3]. TIs are characterized by geometry parameters obtained from various measurement systems, among which the longitudinal level (LL), especially the long wavelength, is a particularly important parameter for assessing the track geometry quality of a track’s longitudinal profile. Nowadays, to ensure operational safety and to enhance comfort during rail travel [4,5], especially for high-speed railway (HSR) networks, railway administrations and companies around the world are conducting track geometry measurements (TGMs) of their rail lines utilizing track geometry cars (TGCs) or track surveying trolley (TSTs) [6,7,8,9,10,11,12]. TGCs can be divided into inertial measurement systems (IMSs) [13] and chord measurement systems (CMSs) [14] according to the type of measurement system. A CMS describes LL by measuring the change in vector distance within a certain chord length, such as 10 m, 20 m, or 40 m, including mid-chords [15] and asymmetric chords [14]. Currently, IMSs are widely used for TGMs, which are based on vertical acceleration variations to determine the LL and involve describing LL mainly through different wavelength ranges [7,10]. The results from these two measurement systems can be transformed within a certain range [10,16]: for example, through the process of inverse filtering [14,17]. Additionally, TSTs, commonly employed using manual and lightweight trolleys [18], are another primary method for obtaining TIs [19,20]. TSTs are typically coordinate measurement systems [18,21]. TSTs perform by measuring the geometry deviations between a track’s current state and the design reference, evaluating the LL through offsets at various chord spans [10,22]. The geometrical condition of a railway can gradually deteriorate [23] and exceed the limits of warning or maintenance [24] after a long period of operation, as well as due to the effects of the natural environment, requiring different maintenance operations [25] to restore its geometrical condition or repair rail defects in accordance with the standards [26,27,28,29,30].

Particularly noteworthy is that the long-wavelength quality of LL for HSR is a key concern for infrastructure maintenance and repair at present [31]. Consequently, scholars and engineers have carried out extensive and thorough research on improving track regularities from various perspectives. At the design level, consideration is given to the inherent standard deviations of a track [32,33], and various transition curves (TCs) are attempted [34]. Regarding operation and maintenance, more focus is placed on providing optimized precise-tamping [35] or fine-adjustment [36] solutions, as well as on the reconstruction of track longitudinal profiles [37]. The existing research findings advance our comprehension and knowledge base of track regularity and further furnish a scientific reference for the diagnosis of track defects.

The results from TGCs provide direct indications of the LL. However, due to drawbacks such as mileage errors [38,39], waveform distortions [22], and high costs, their results are not directly applied to track fine-adjustment operations. In daily operation and maintenance, management personnel customarily employ the findings from TGCs as the cornerstones for their assessments and decision-making [40]. These results are then supplemented with the precise deviations from TSTs to meticulously re-evaluate pivotal sections of the track. This dual-process approach facilitates a thorough evaluation of track regularity quality and serves as a guiding principle for fine-adjustment operations [26].

In China, the 120 m cutoff wavelength track LL of HSR exceeds by 5 mm and 7 mm as I-level and II-level over-limit respectively, and the vehicle body vertical acceleration exceeds by 0.1 g as II-level over-limit [41]. Actually, frequent II-level over-limit alarms of vertical acceleration in these regions pose a challenging issue to resolve [37]. An analysis of TGMs along operational lines, coupled with daily maintenance practices, reveals irregularities near vertical curves (VCs) reminiscent of track deformation [40]. However, further experimental analyses have identified significant discrepancies between the output vertical long-wavelength irregularities derived from TGCs and TSTs, particularly at the beginning and ending points of a VC (BEVC). This raises the question of whether the observed discrepancies imply the actual presence of vertical irregularities or not, and which set of results, those from TGCs or TSTs, might be inaccurate. If TSTs truly reflect track geometry, indicating the absence of vertical irregularities, then identifying the causes of irregularities from TGCs becomes necessary. Conversely, if TGCs accurately portray track geometry, research into methods to mitigate these vertical irregularities becomes essential. To this end, the aim of this research is to investigate the factors underlying the disparities in vertical long-wavelength irregularities observed at the BEVC from TGCs, and to propose solutions. The resultant research findings are thus theoretically and technologically useful for the design, construction, and maintenance of HSR.

This paper is organized as follows: Section 2 analyses the specific characteristics of the track vertical irregularities in TGCs. A simulation model for an IMS is designed and validated in Section 3. The quantitative relationship between SLIs and longitudinal profiles of HSR is discussed in Section 4. Methods to mitigate SLIs and validation experiments are presented in Section 5.

## 2. The Characteristics of Vertical Irregularities

**Figure 1:**
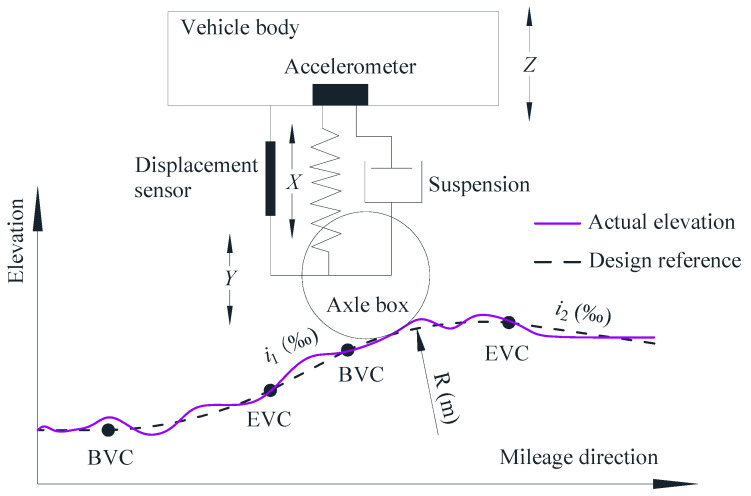
Schematic representation of a railway track VC and TGCs based on an IMS.

Figure 1 presents a schematic diagram of a VC and TGCs based on an IMS. A VC with a radius *R* (m) connects two different gradients, *i*_1_‰ and *i*_2_‰, between the beginning point (BVC) and the ending point (EVC), facilitating uphill and downhill transitions. The VC at the top of the hill is termed a summit curve, while the VC at the bottom of a hill is termed a sag curve. Currently, TGCs are predominantly conducted using IMSs, which utilize inertial measurements and digital filtering to output the LL based on changes in vertical acceleration [6,9,13]: refer to Figure 1 for details. The LL irregularities *Y* of a track are determined using the formula presented below.

(1) $$Y=Z+X=\iint a_{v}dtdt+X$$

where *Z* represents the vertical movement of the vehicle body, calculated via double integration of the vertical acceleration *a_v_*, and *X* indicates the relative displacement between the vehicle body and the axle box, which is directly measured with a displacement sensor. Accelerometers are utilized to detect low-frequency, long-wave irregularities, whereas displacement sensors are employed for measuring high-frequency, short-wave irregularities. Theoretically, IMSs are capable of detecting the LL over an infinite range of wavelengths, yet in practice, attention is paid only to those that impact the safety and comfort of operation. Consequently, high-pass filters are typically applied in IMSs to generate outputs of LL across various wavebands, such as the typical wavebands W1, 1.5 m to 150 m; W2, 1.5 m to 70 m; and W3, 1.5 m to 25 m.

TSTs are utilized by using a manual and lightweight trolley as the carrier, which is based on a track control network, and employing a total station (Geometric Benchmark, GB) or inertial navigation unit (Inertia Benchmark, IB) [42] to precisely measure the track coordinates and elevation. These measurements are subsequently analyzed computationally to ascertain the precise geometric deviations of the track relative to its design reference.

**Figure 2:**
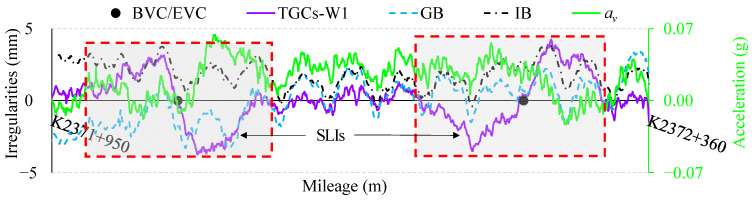
Long-wavelength deformation in VC section.

Typically, the IMS output of LL, particularly in the long-wave wavebands, aligns well with the deviations calculated by TSTs, and the discrepancies between them generally fall within an acceptable range. Nonetheless, through an analysis of TGMs, coupled with daily maintenance practices, notable discrepancies have been discerned to exist between TGCs and TSTs in the vicinity of the BEVC, and these discrepancies exhibit the characteristics of universality and repeatability. Figure 2 presents an illustrative example of track vertical irregularities from TGCs and TSTs for a ballastless track in the VC section of a GSG HSR. The horizontal axis is the mileage, and the vertical axis is the vertical irregularities and vertical acceleration of the vehicle body. TGCs-W1 denotes the vertical irregularities provided by TGCs within waveband W1, typically referred to as long-wave. In contrast, GB and IB represent the vertical deviations measured from TSTs based on a geometric benchmark (utilizing Amberg GRP 1000 s) and an inertial benchmark (utilizing Railway-Helper GJY-TW-RB-0), respectively. *a_v_* represents the vertical acceleration of the vehicle body.

Figure 2 reveals that the relative relationships of the track’s vertical deviation waveforms from the two TSI modalities are fundamentally aligned. However, significant disparities are observed in the output between TGCs and TSTs around the BVC/EVC, where TGCs-W1 undergoes severe deformation, resembling large S-shaped long-wave irregularities (SLIs). Additionally, the vertical acceleration of the vehicle body in this section also increases significantly. In other sections, however, similar discrepancies are not found.

### 2.1. Vertical Long-Wave Irregularities

To further explore the characteristics of SLIs, we discuss a thorough investigation of the track geometry parameters collected periodically with TGCs in the GSG HSR for a period of one year, covering more than 200 km of both the up and down tracks. The railway line features a ballastless concrete slab track bed, designed for a speed of 350 km/h, equipped with seamless steel rails that weigh 60 kg/m, and has a maximum gradient of 30‰, along with 106 VCs.

**Figure 3:**
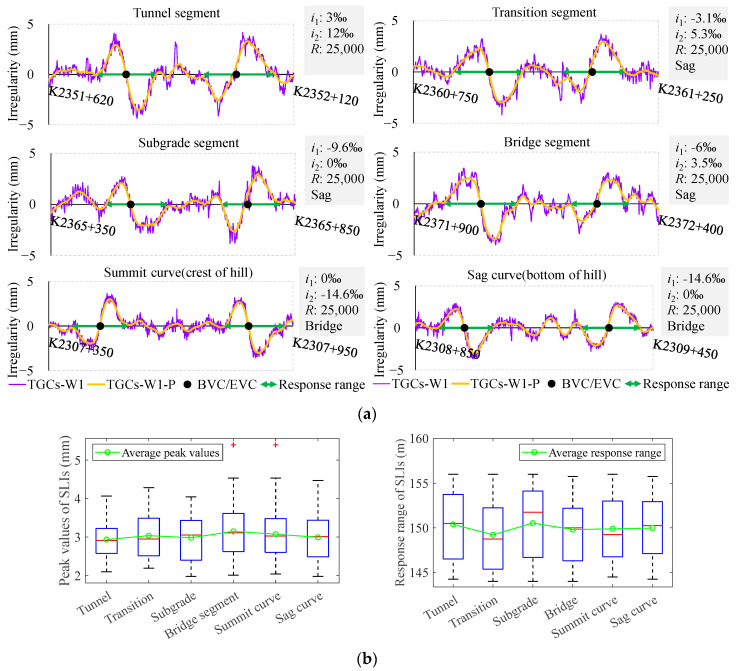
SLIs with different subgrade foundations and locations: (**a**) examples of SLIs with different subgrade foundations and locations; (**b**) peak values and response range of SLIs according to different subgrade foundations and locations using box-plots.

**Figure 4:**
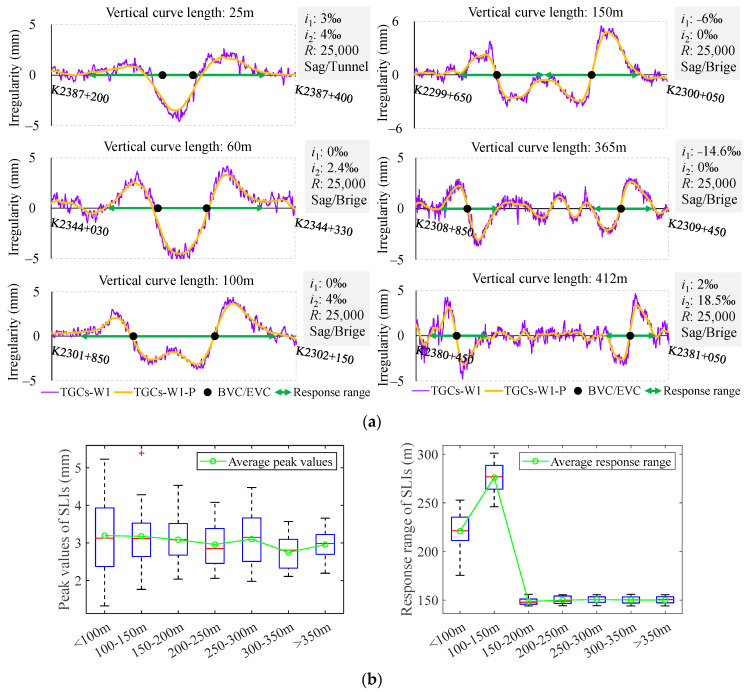
SLIs with VC lengths ranging from 25 m to 412 m: (**a**) examples of SLIs with different VC lengths; (**b**) peak values and response range of SLIs according to different VC lengths using box-plots.

**Figure 5:**
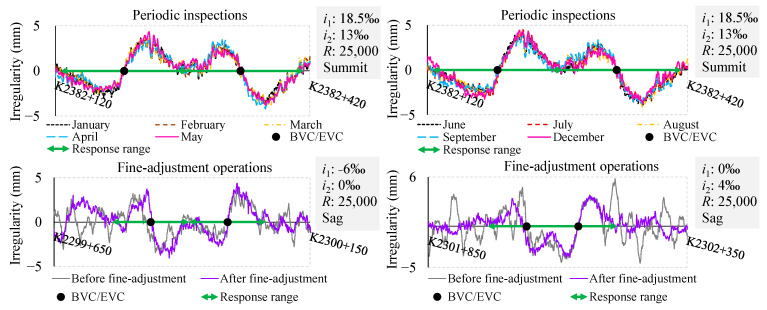
SLIs at different inspection times.

To eliminate the effects of measurement errors and ultra-short-wavelength irregularities of the track, a filtering pre-processing step is applied to the vertical irregularities output by the TGCs, which are denoted as TGCs-W1-P, TGCs-W2-P, and TGCs-W3-P in the following figures. Subsequently, VCs are to be classified according to the subgrade foundations (in Figure 3), their positions (in Figure 3), lengths (in Figure 4), the times of inspections (in Figure 5), and the fine-adjustment operations (in Figure 5). This classification will facilitate an analysis of the SLIs’ peak values and response ranges, where the peak value is defined as the amplitude from zero to the peak value. The response range is identified based on the beginning and ending points of waveform deformation and is indicated with double arrows on the chart. In consideration of the difference in track loading exerted by TGCs and TSTs, conducting an initial investigation on whether the SLIs are correlated with the types of subgrade foundations is pertinent, as shown in Figure 3.

The initial quartet of diagrams in Figure 3a presents a series of examples illustrating the SLIs in four prevalent types of subgrade foundations: a tunnel segment, a transition segment, a subgrade segment, and a bridge segment. As shown in Figure 3b, extensive statistics indicate that the SLIs maintain a fundamental uniformity across these different subgrade foundations, with an average peak value of 3 mm and an average response range of 150 m. Following this, we further investigate the locations of VCs at the crest and bottom of a hill. When the VC is located at the crest or at the bottom, its concavity and convexity are inverted. As illustrated in the final pair of diagrams in Figure 3, the SLIs persist irrespective of the VC’s location, maintaining a consistent peak value and response range, with only a corresponding shift in direction. Another critical factor under consideration is the length of the VCs. Subsequent investigations have thus been conducted on the variation patterns of SLIs concerning a VC spanning lengths from 25 m to 412 m.

As shown in Figure 4, when the length of the VC is less than 150 m, the superimposition of overlapping segments is observed to result in alterations to both the peak value and response range. Conversely, when the length exceeds 150 m, the S-shaped waveform shifts towards both ends and reverts to its pristine form, indicating an average peak value of 3 mm and an average response range of 150 m.

The aforementioned investigations have focused on SLIs collected at different mileage locations within a single batch. Moving forward, this study will further consider conducting a comparative analysis of periodic inspections, including interventions for track fine-adjustment operations. After collecting and organizing nearly a year’s worth of output data from monthly TGCs, the SLIs were aligned based on mileage and superimposed, as depicted in the first two images of Figure 5. Notably, distinct SLIs are present in the waveforms around the BVC/EVC points for each month. Moreover, the waveforms are in accordance with each other, with variations of less than 1 mm. As depicted in the last two images of Figure 5, track fine-adjustment operations evidently did not mitigate the SLIs. This further indicates no discernible correlation between the SLIs and the times of inspections or the implementation of fine-adjustment operations.

Based on observations and validations through substantial datasets from the TGCs, we have identified notable SLIs at the BEVC of this HSR, exhibiting a symmetrical pattern. Notably, these SLIs are not significantly associated with common subgrade foundations, the locations of VCs (at the crest or bottom of a hill), the times of inspections, or fine-adjustment operations. The peak value and response range of these SLIs remain essentially stable.

### 2.2. Other Wavebands

**Figure 6:**
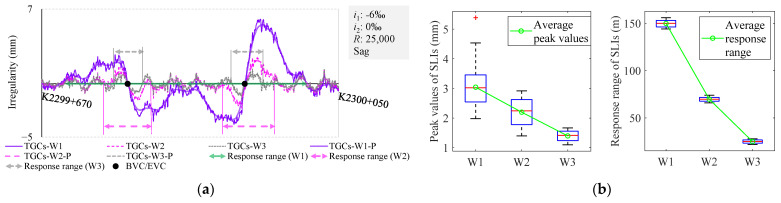
Track vertical irregularities derived from different wavebands: (**a**) examples of S-shaped irregularities in different wavebands; (**b**) peak values and response range of SLIs according to different wavebands using box-plots.

From the perspective of a long-wave analysis, SLIs are distinctly evident in TGCs-W1. It is thus logical to contemplate whether similar patterns exist in other wavebands. Apart from waveband W1, TGCs are equipped to identify waveforms representing the LL in additional wavebands, including W2 and W3. Figure 6 illustrates a comparison of track vertical irregularities across three distinct wavebands. TGCs-W2 and TGCs-W3 denote the irregularities derived from wavebands W2 and W3.

The waveforms are noted to exhibit consistent S-shaped irregularities around the BEVC. Further research reveals that the peak value and response range of these S-shaped irregularities are intimately correlated with the cutoff wavelength of the wavebands, as shown in Figure 6b. Specifically, the average peak values are 3 mm, 2.2 mm, and 1.4 mm, with corresponding average response ranges of 150 m, 70 m, and 25 m, respectively.

In general, the S-shaped irregularities from the TGCs exhibit a notable universality, particularly in the vicinity of the BVC and EVC points. The characteristics of the S-shaped irregularities are closely associated with the cutoff wavelength of the wavebands. Specifically, the longer the cutoff wavelength, the greater the peak value and response range of the S-shaped irregularities, and vice versa. Moreover, SLIs have been found to be invariant to certain verified factors, including the type of subgrade foundation, the location and the length of the VC, and the times of inspection. This constancy suggests that SLIs are characterized by non-randomness, repetitiveness, and systematic errors in terms of direction and magnitude.

## 3. Simulation Models and Validation

As discussed at the beginning of Section 2, TGCs based on IMSs involve delineating the spatial curvature of a rail top surface within an inertial reference frame through integrated operations [43]. Subsequently, high-pass filtering has been applied to extract specific wavelength components that are indicative of the LL. Recognizing the systematic error traits inherent in SLIs, a thorough and nuanced analysis was subsequently conducted from the perspective of simulation IMSs.

### 3.1. Simulation Models

**Figure 7:**
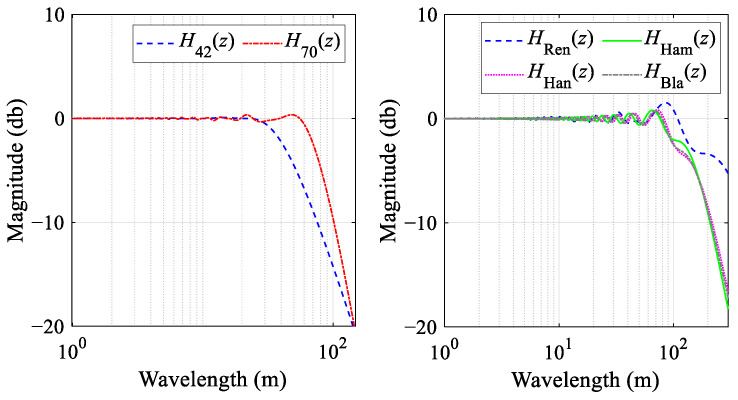
Amplitude–frequency characteristics of system function.

IMSs undergo digital filtering in the spatial domain, utilizing a track spatial curve determined according to acceleration integration as the primary input signal [22,43]. To maintain the integrity of the waveform’s spatial relative positioning through the filtering process, the filter must have linear phase–frequency characteristics. This requirement ensures that the spatial alignment of the waveform is preserved, thereby guaranteeing the fidelity and reliability of the track irregularities post filtering. Within the realm of railway inspections and assessments, the system functions *H*_42_(*z*) and *H*_70_(*z*) for IMSs, which correspond to the 42 m and 70 m cutoff wavelengths, respectively, play pivotal roles in the analytical process. These system functions are the cornerstones of the digital filtering mechanisms employed to process spatial curve data [17,44]. The expressions for these system functions are as follows:

(2) $$H_{42}(z)=z^{-123}-\frac{1}{83^{3}}{\left[\frac{1-z^{-83}}{1-z^{-1}}\right]}^{3}$$

(3) $$H_{70}(z)=1-1.036{\left[\frac{z^{80}-z^{-80}}{160(1-z^{-1})}\right]}^{2}+0.036{\left[\frac{z^{20}-z^{-20}}{40(1-z^{-1})}\right]}^{2}-0.25\left[\frac{1}{321}\left(\frac{z^{160}-z^{-161}}{1-z^{-1}}\right)-\frac{1}{561}\left(\frac{z^{280}-z^{-281}}{1-z^{-1}}\right)\right]$$

where *z* is a complex variable. Equation (2) comprises a parallel combination of an all-pass filter and a third-order low-pass filter, and Equation (3) comprises a parallel combination of two triangular windows and two rectangular windows. The amplitude–frequency characteristics are depicted on the left side of Figure 7.

Currently, interest in the irregularities of long-wave components, which are critical for maintaining the safety and comfort of an HSR, has increased. Despite that, the existing body of literature appears to be deficient in studies that detail filtering systems with a cutoff wavelength of 150 m. This dearth of research has prompted an investigation into the design of specialized long-wavelength filters, with the objective of achieving a more profound understanding and investigation of the factors leading to SLIs. Building upon the existing system functions *H*_42_(*z*) and *H*_70_(*z*), the proposed design strategy envisions the extraction of long-wave components as a primary objective, which is achieved by employing a low-pass filter that serves as the base window for the initial phase of the process. Subsequently, to mitigate the side-lobe leakage emanating from the base window, a refinement technique is introduced. This technique involves the parallel application of a triangular window or a band-stop filter, which effectively suppresses the side lobes and enhances the filter’s performance by reducing unwanted signal components.

In the forthcoming section, we introduce a duality of methodologies dedicated to the development of a filter model with a cutoff wavelength of 150 m for simulating IMSs. The initial approach entails a systematic adjustment of the window length and the coefficients of the associated window function, guided by the principles underpinning the *H*_70_(*z*) system function. This iterative process continues until the amplitude–frequency characteristics and the cutoff wavelength of the filter are in strict accordance with the predefined specifications. Through a series of optimization simulations, involving a meticulous tuning process, a filter system function model, denoted as *H*_Ren_(*z*), is derived. This function encapsulates the desired properties essential for an accurate simulation of an IMS. The filter system function model *H*_Ren_(*z*) is presented in Equation (4), and its amplitude–frequency characteristics are presented in the right panel of Figure 7.

(4) $$H_{Ren}(z)=1-1.01{\left[\frac{z^{240}-z^{-240}}{480(1-z^{-1})}\right]}^{2}+0.01{\left[\frac{z^{20}-z^{-20}}{40(1-z^{-1})}\right]}^{2}-0.55\left[\frac{z^{140}-z^{-141}}{141(1-z^{-1})}-\frac{z^{480}-z^{-481}}{481(1-z^{-1})}\right]$$

An alternative approach is also proposed, which employs the use of window functions to achieve the desired filtering characteristics. In this approach, the cutoff frequencies that define the transition from the passband to the stopband are represented by *w*_p_ and *w*_s_, respectively. The construction of an approximate frequency response function, denoted as *H*_d_(*e^jw^*), is established as follows:

(5) $$H_{d}(e^{jw})=\left\{ \begin{array}{l} e^{-jw\varepsilon }\;\;\;w_{c}\leq |w|\leq \pi \\ 0\;\;\;\;\;\;\;\;0\leq |w|\leq w_{c} \end{array}\right.$$

where *w*_c_ = (*w*_p_ + *w*_s_)/2 and *ε* = (*N* − 1)/2, while *N* denotes the dimension of the window function. Notably, *N* is contingent upon the parameter *B*_t_, signifying the width of the transition band inherent to the window function. This dependency is governed by the following relationship:

(6) $$\left\{ \begin{array}{l} B_{t}\leq w_{p}-w_{s}\\ N=2k+1(k\in Z) \end{array}\right.$$

Subsequently, the unit impulse response function, denoted as *h*(*n*), of the filter is derived.

(7) $$h(n)=\frac{1}{2\pi }\int_{-\pi }^{\pi }H_{d}(e^{jw})e^{jwn}dww(n)=\left\{\delta (n-\varepsilon )-\frac{sin[w_{c}(n-\varepsilon )]}{\pi (n-\varepsilon )}\right\}w(n)$$

Herein, *δ*(*n* − *ε*) represents the unit pulse sequence, and *w*(*n*) signifies the diverse window functions that are integral to the filter design. Thus, the primary filter system function can be articulated as follows:

(8) $$H_{m}(z)=\sum_{n=0}^{N-1}h(n)z^{-n}$$

Given that an excessively long length of window function *N* can impose considerable computational burdens and the prolonged slope of longitudinal profiles significantly affects the output sequence, a two-step approach is adopted. Initially, a rectangular window with width *N*_0_ is cascaded with the primary filter system to extract the long-wave trend component from the output of *H*_m_(*z*). Subsequently, the window is integrated in parallel with the primary filter to amend the output of the system function *H*_m_(*z*). Consequently, the formulation for the long-wave system function model *H*(*z*) is organized as follows:

(9) $$H(z)=H_{m}(z)\left[1-\frac{1-z^{-N_{0}}}{N_{0}(1-z^{-1})}\right]$$

To accurately simulate the long-wave output of an IMS, three distinct window functions are employed: the Hanning, Hamming, and Blackman windows. These window functions are sequentially substituted into Equation (7) to derive three separate system function models, marked as *H*_Han_(*z*), *H*_Ham_(*z*), and *H*_Bla_(*z*), which correspond to the simulated IMS long-wave filtering. The amplitude–frequency responses of these functions are graphically depicted in the right panel of Figure 7.

### 3.2. Experimental Validation

**Figure 8:**
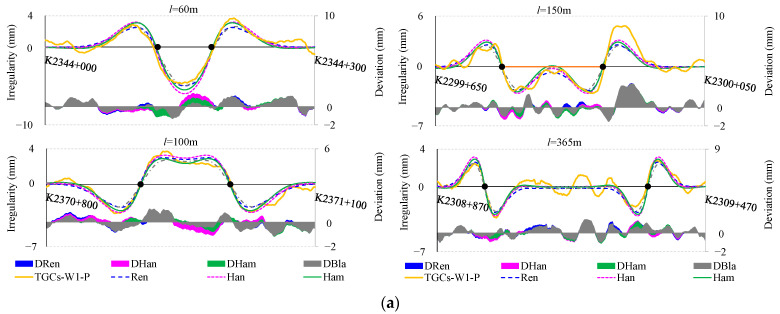

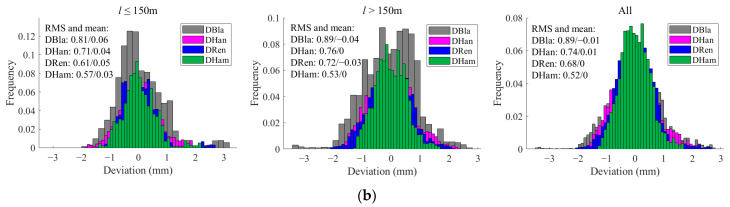
Vertical irregularities simulated individually with four distinct simulation models: (**a**) comparison and deviation between simulated and actual waveforms; (**b**) frequency histogram of deviations based on the length of the VCs.

In the following section, we critically evaluate the accuracy and feasibility of the four previously delineated simulation models through multiple dimensions. The initial validation approach employs the longitudinal profiles of the GSG HSR design as an input sequence to simulate the inertial measurement process. Specifically, this input sequence is sequentially fed into *H*_Ren_(*z*), *H*_Han_(*z*), *H*_Ham_(*z*), and *H*_Bla_(*z*) for corresponding processing. Subsequently, the simulated outputs are compared with those of TGCs-W1-P. To achieve a validation that is both robust and persuasive, deliberating on the selection of VC sections with varying radii (*R*); lengths (*l*); and locations, such as sag or summit curves, is essential. As presented in Figure 8, Ren, Han, Ham, and Bla, respectively, represent the track vertical irregularities simulated by *H*_Ren_(*z*), *H*_Han_(*z*), *H*_Ham_(*z*), and *H*_Bla_(*z*). Concurrently, DRen, DHan, DHam, and DBla represent the deviation values for these simulated outcomes and TGCs-W1-P. Figure 8a presents a comparison and deviation examples between simulated and actual waveforms for different VC lengths. Figure 8b offers a frequency histogram of deviations based on the length of the VCs, divided into segments for VCs of *l* ≤ 150 m, VCs of *l* > 150 m, and all VCs, along with the root-mean-square (RMS) and mean values for the four model deviations, all labeled on the histogram.

According to Figure 8a, the quartet of simulation outputs show a striking consistency: notable SLIs are evident in the vicinity of the BVC/EVC points, while maintaining a constant output at zero within the other ranges. From the deviation frequency histogram in Figure 8b, all four models show good simulation effects, with average deviations close to 0 and RMS values below 0.9. However, it is also evident that the Ham results are closer to the actual waveforms measured by the TGCs, with an average deviation close to 0 and an RMS value of about 0.52. Therefore, the system function model *H*_Ham_(*z*) is posited to provide a more accurate and effective simulation of the IMS than its counterparts.

**Figure 9:**
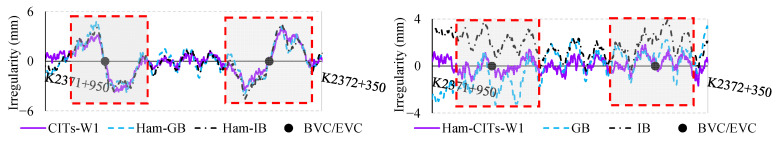
Example of further validation by applying *H*_Ham_(*z*).

Subsequently, the accuracy and validity of *H*_Ham_(*z*) for simulating an IMS are further verified from two additional perspectives, as shown in Figure 9. Firstly, actual elevation is procured utilizing two distinct TST modalities: the GB and IB modes. These datasets are subsequently subjected to the filtering processes, denoted as Ham-GB and Ham-IB. As illustrated in the left panel of Figure 9, the outcomes reveal the conspicuous presence of SLIs still in Ham-GB and Ham-IB, and exhibit an aggregate deviation of less than 1 mm from TGCs-W1, as shown in the red dash line boxes. Secondly, efforts have been directed towards eliminating SLIs in TGCs-W1 through the application of *H*_Ham_(*z*). As illustrated in the Ham–TGCs-W1 curve in the right panel of Figure 9, these SLIs are effectively eliminated in the vicinity of the BEVC. Moreover, a significant level of correspondence is observed between Ham-TGCs-W1 and GB or IB, as shown in the red dash line boxes. These findings serve to further substantiate the accuracy and feasibility of simulating long-wave irregularities from IMSs utilizing the system function model *H*_Ham_(*z*).

Following this thorough comparative analysis of vertical irregularities from TGCs and TSTs, coupled with a simulation study of the long-wave filter system employed in IMSs, SLIs are deduced to be a consequence of the filtering system’s specific response to the input VCs. Generally, a railway VC transitions directly from a slope without intermediary TCs. As TGCs pass through the BEVC, abrupt changes occur in the vertical acceleration. Consequently, TGCs that based on the IMS output vertical track irregularities, manifesting as SLIs. The combination of design-induced irregularities and track deformations makes these areas susceptible to violations of vertical acceleration and irregularity thresholds. The SLIs observed during a TGC’s operation also accurately reflect the characteristics of changes in the vehicle body’s vertical acceleration. Owing to variations in measurement reference, no design-induced irregularities are accounted in TSTs. Therefore, these SLIs are not attributed to geometric deformation from the design reference. Instead, a deficient longitudinal profile design is what leads to the track vertical irregularities observed in TGCs.

## 4. Quantitative Relationship

Merely qualitative discussions are clearly insufficient to meet practical requirements. Routine maintenance is much more interested in quantitative analyses of TIs. Therefore, referring to the maintenance rules in [45], here, we use a maximum gradient for the HSR of 30‰ and a minimum radius for the VC of 15,000 m. Taking the VC radius *R* (m) and the gradient difference Δ*i* (‰) between adjacent slopes as variables, four sets of longitudinal profiles (Groups A to D) have been combined with the parameters listed in Table 1.

Specifically, in Group A, 12 longitudinal profiles were designed, with a radius set at the common value of 25,000 m and a gradient difference that ranges from 8‰ to 30‰, incremented by 2‰. For Groups B to D, 21 longitudinal profiles were designed each, with the radius varying from 10,000 m to 30,000 m, incremented by 1000 m, and the gradient difference set at 10%, 20%, and 30%, respectively. Subsequently, these various longitudinal profiles served as inputs to *H*_Ham_(*z*), facilitating an investigation into the mathematical relationship between SLIs and longitudinal profiles.

**Figure 10:**
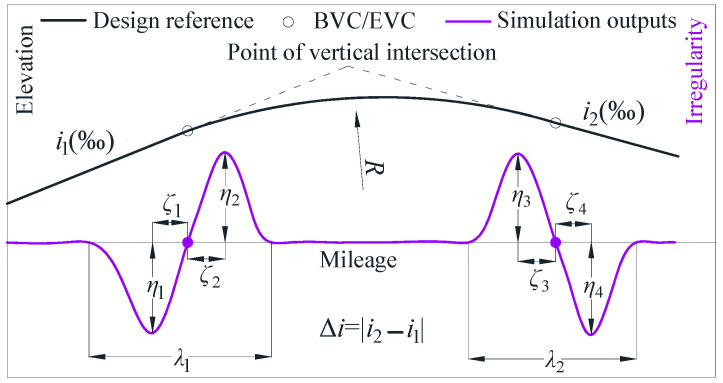
The characteristic parameters of SLIs.

**Figure 11:**
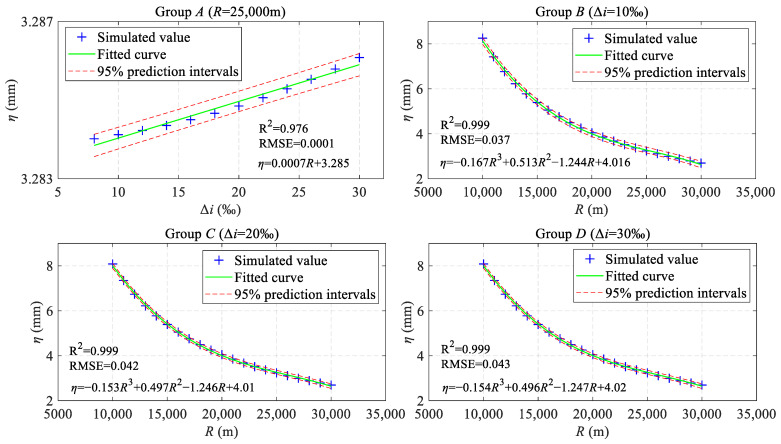
The characteristic parameters of the SLIs’ regression relationships between the peak value *η* and the gradient difference Δ*i*, as well as the radius *R*.

**Figure 12:**
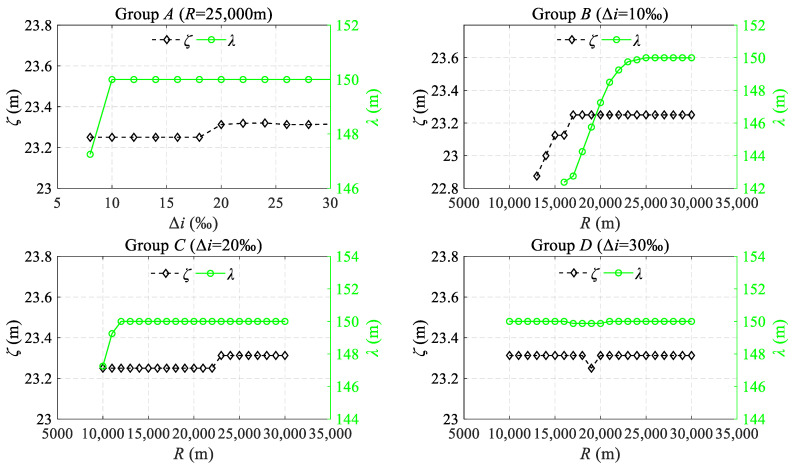
The relationship of the change in *ζ* and *λ* with the gradient difference Δ*i* and the radius *R*.

As depicted in Figure 10, for each longitudinal profile, the SLIs were characterized by three distinct categories of characteristic parameters: the peak value *η_i_* (*I* = 1, 2, 3, 4), the horizontal distance from the peak point to the BVC/EVC point *ζ_i_* (*I* = 1, 2, 3, 4), and the response range *λ_i_* (*I* = 1, 2). The longitudinal profiles from Groups A to D were fed into *H*_Ham_(*z*) for a statistical evaluation of their characteristic parameters. Building upon these quantifications, the regression relationship among the average values of the three characteristic parameters—*η*, *ζ*, and *λ*—and Δ*i* and *R* values was analyzed, as shown in Figure 11 and Figure 12.

As shown in Figure 11, a linear correlation exists between the peak value *η* and the gradient difference Δ*i* in Group A. The coefficient of determination (R^2^) is 0.976, and the root-mean-square error (RMSE) is 0.0001. The specific regression equation is annotated on the figure for reference (the same applies to subsequent figures). This correlation indicates that when the radius *R* is fixed and the gradient difference Δ*i* is gradually increased from 8‰ to 30‰, the linear functional relationship between *η* and Δ*i* is significant. Nonetheless, the fluctuation In *η* is confined with a narrow range, approximately 3.284 ± 2.8 × 10^−4^ mm at a 95% confidence level. This finding suggests that the peak value of SLIs is marginally influenced by the gradient difference. For Groups B to D, guided by the R^2^ and RMSE, cubic polynomials were adopted for fitting (*R* was centered to zero and scaled to a unit of standard deviation). Evidently, the peak value *η* decreases as the radius *R* increases. Specifically, when *R* increases from 10,000 m to 20,000 m, *η* decreases significantly, dropping from 8.254 mm, 8.085 mm, and 8.084 mm, respectively, to 4.042 mm. In contrast, when *R* increases from 20,000 m to 30,000 m, the decrease in *η* tends to level off, from approximately 4.042 mm to 2.695 mm. Upon comprehensive analysis, the peak values of SLIs can be inferred to be significantly influenced by the radius of the VC, particularly when the radius is less than 20,000 m.

As shown in Figure 12, for Group B, where *R* is less than 17,000 m, the value of *ζ* varies in accordance with the increase in *R*. In other instances, *ζ* is essentially fixed at around 23.3 m. This demonstrates a stable and predictable pattern in the waveform’s peak location of SLIs. Considering the discernibility of the peak points, this facilitates the convenient establishment of a correlation between the inspection waveform and the corresponding onsite location. Regarding the response range *λ*, in Group A*,* where Δ*i* is less than 10‰, *λ* exhibits a rapid increase with the expansion of Δ*i*. This trend is mirrored in Group B and C, where *R* is less than 23,000 m and 12,000 m. However, when Δ*i* or *R* deviates from the aforementioned range, the value of *λ* remains essentially invariant at 150 m, corresponding to the cutoff wavelength of W1. Additional research demonstrates that Δ*i* in Group A or R in Groups B and C taking small values can lead to the length of the VC being less than 150 m, and the S-shaped waves at the BEVC may superpose on each other. This leads to changes in *ζ* (less than 23.3 m) and *λ* (less than 150 m). In contrast, once the length of the VC exceeds 150 m, the S-shaped wave returns to its pristine form, with its response range fixed at 150 m. Indeed, as previously analyzed, the response range of the S-shaped wave is influenced by the cutoff wavelength of the IMS.

Upon a comprehensive review of the conducted IMS simulation experiments, several pivotal conclusions can be distilled. When the radius is fixed, the peak value of SLIs is less affected by the gradient difference. Conversely, under conditions where the gradient difference is constant, the peak value of SLIs is significantly influenced by the radius, decreasing as the radius increases. Furthermore, at distances of approximately 23.3 m from the BVC and EVC, the SLIs reach their peak and then gradually attenuate towards zero on both sides. Additionally, as the length of the VC exceeds 150 m, variations in the gradient difference and radius cease to impact the response range and the location of its peak. This implies that SLIs are a distinctive response pattern elicited by IMS filters.

## 5. Method for Mitigating SLIs

**Figure 13:**
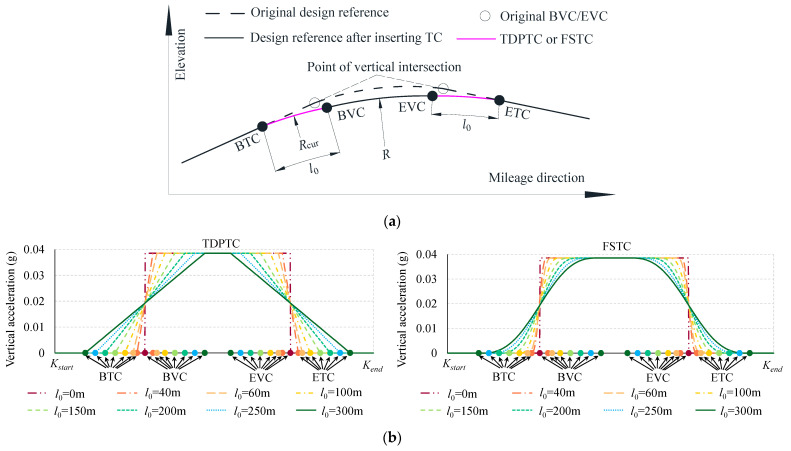
Optimized longitudinal profile and simulation of vertical acceleration: (**a**) schematic representation of a VC with an added TC; (**b**) the variation in simulated *a_v_* (*V* = 350 km/h, *R* = 25,000 m).

The preceding section has indicated that when the radius reaches 30,000 m, the peak value of SLIs still remains at approximately 2.7 mm. Considering the constraints of adjustable quantities for ballastless tracks and the challenges associated with maintenance, persisting in managing vertical irregularities by increasing the radius is infeasible. Given that SLIs are induced by abrupt changes in vertical acceleration, the key to mitigating these irregularities lies in eliminating abrupt changes in acceleration. In both HSRs and high-speed maglev lines, third-degree parabolic transition curves (TDPTCs) and full-wave sinusoidal transition curves (FSTCs) are extensively applied between tangent and circular sections. Subsequently, we consider adding a TDPTC or an FSTC at both ends of the designed VC to smoothly connect gradients and VCs. The parametric equations for the TDPTC and FSTC are as follows:

(10) $$\left\{ \begin{array}{l} y=\frac{x^{3}}{6Rl_{0}}\;\;\;(\mathrm{TDPTC})\\ y=\frac{l_{0}^{2}}{R}\left[\frac{1}{6}{\left(\frac{x}{l_{0}}\right)}^{3}-\frac{1}{4\pi^{2}}\left(\frac{x}{l_{0}}\right)+\frac{1}{8\pi^{3}}\sin\left(\frac{2\pi x}{l_{0}}\right)\right]\;\;\;\;\;(\mathrm{FSTC}) \end{array}\right.$$

where *R* is the radius of the VC, and *l*_0_ is the length of the TC. As shown in Figure 13a, following the increasing mileage, the design reference sequentially passes through the beginnings of the transition curve (BTC) and vertical curve (BVC), the end of the vertical curve (EVC), and the transition curve (ETC). The variations in the vertical acceleration *a_v_* of the vehicle body, with *l*_0_ ranging from 0 to 300 m, are simulated using the point mass acceleration (PMA) method [46]. The acceleration is calculated using the following equation:

(11) $$a_{v}=\frac{V^{2}}{12.96R_{cur}}$$

where *V* is the design speed, set at 350 km/h, and *R*_cur_ is the radius of the curvature at any point on the design reference. With *l*_0_ gradually extending from 0 to 300 m, a simulation experiment was conducted on the vertical acceleration experienced by the train body as it traverses, as illustrated in Figure 13b.

Without a TC (*l*_0_ = 0), *a_v_* undergoes an abrupt change from 0 to a maximum value of 0.04 g at the BVC point and remains constant thereafter until the EVC point, where it then abruptly drops from 0.04 g back to 0. However, when TCs are introduced, *a_v_* gradually increases from 0 to 0.04 g between the BTC and BVC points, remains constant at this maximum value, and then gradually decreases from 0.04 g back to 0 between the EVC and ETC points. In contrast, *a_v_* demonstrates a linear trend for TDPTCs, but for FSTCs, it follows a curvilinear pattern, with slow changes at the ends and rapid changes in the middle. After the addition of TCs, the abrupt changes in vertical acceleration were eliminated. Following this, the impact on the SLIs in TGCs will be further verified.

**Figure 14:**
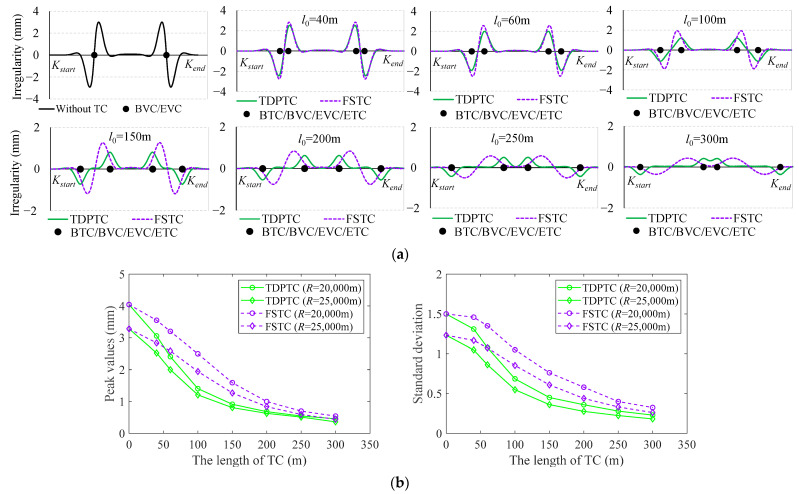
Comparison of SLIs after the addition of TDPTCs and FSTCs: (**a**) comparison of SLI waveforms after the addition of TDPTCs and FSTCs (*R* = 25,000 m); (**b**) changes in the peak values and SD of SLIs after the addition of TDPTCs and FSTCs (*R* = 20,000 m, *R* = 25,000 m).

Referring to the code for the design of an HSR [45], the minimum radius of the VC is 20,000 m, 25,000 m, and 25,000 m for design speeds of 250 km/h, 300 km/h, and 350 km/h, respectively. For these two radii, consideration is given to adding TDPTCs and FSTCs at the BEVC, with *l*_0_ ranging from 0 to 300 m. After adding two types of TCs, the simulation comparison of the SLIs, as well as the changes in the peak values and standard deviation (SD) of the SLIs, is shown in Figure 14.

As shown in Figure 14a, as the length of the TCs increases, the SLIs clearly diminish progressively. However, for TDPTCs and FSTCs, there are distinct differences in the waveforms of the simulated outputs. Specifically, for TDPTCs, as the length of the TCs increases, the waveforms at both ends of the TCs gradually separate, exhibiting a semi-S shape. In the case of the FSTCs, the waveforms consistently display an S shape.

As shown in Figure 14b, without inserting a TC (*l*_0_ = 0 m), the peak values and SD of SLIs exceed 3 mm and 1.2, respectively. Conversely, as *l*_0_ expands to 200 m, the peak values drop below 1 mm, and the SD is below 0.6. As *l*_0_ reaches 300 m, the peak values for TDPTCs and FSTCs are further diminished to negligible levels of 0.5 mm and 0.3 mm, respectively, and the SD is below 0.6. Overall, the longer the TC, the smaller the peak values and SD of the SLIs, and a TDPTC has better smoothing properties relative to a FSTC. Therefore, implementing a TDPTC is recommended for mitigating SLIs at the ends of VCs. Considering that longitudinal profiles currently lack the design of a TC, further on-site engineering comparisons with TGCs will be conducted in subsequent research.

Significant discrepancies between the output vertical irregularities derived from TGCs and TSTs are evidently associated with abrupt changes in vertical acceleration. In summary, adding TCs can eliminate these abrupt changes in vertical acceleration and simultaneously reduce the peak values and SD of SLIs, thereby enhancing track regularity and ride comfort. This is attributed to the typical inclusion of an extended TC in VC designs, which can serve to smoothly connect gradients and VCs.

## 6. Conclusions

This research is grounded in the operational and maintenance practices of an HSR, with a concentrated focus on controlling track geometrical irregularities through periodic TGMs. SLIs were found to be present in the vertical direction from TGCs at the BEVC. Drawing upon extensive on-site surveys and comparative analyses, we have thoroughly explored the sequential characteristics of SLIs associated with railway VCs, and, from the perspective of spatial filtering, designed a long-wave filter for simulating IMSs. Ultimately, a method is proposed in this paper for mitigating SLIs. Some more detailed conclusions have also been obtained, as follows:

There are significant discrepancies between the output of vertical irregularities from TGCs and TSTs, manifesting as SLIs. When the cutoff wavelengths of an IMS are 150 m, 70 m, and 25 m, the average peaks of the SLIs are 3 mm, 2.2 mm, and 1.4 mm, with corresponding average response ranges of 150 m, 70 m, and 25 m, respectively.

SLIs cannot be attributed to geometric deformations of the track. Instead, they are caused by deficiencies in the longitudinal profile design leading to abrupt changes in vertical acceleration.

The gradient difference has a minimal impact on SLIs, whereas the radius of VCs exerts a significant effect. With an increase in radius, the peaks of SLIs decrease markedly. However, once the radius exceeds 20,000 m, its impact on SLIs gradually diminishes.

By adding TCs at both ends of a VC, the curvature radius transitions incrementally from infinity to a set value. Thus, effectively eliminating abrupt changes in the vertical acceleration experienced by high-speed trains and concurrently mitigating SLIs on the track become feasible. Additionally, TDPTCs are recommended to be added at both ends of VCs for mitigating SLIs.

The research findings in this paper are therefore theoretically and technologically useful in the design, construction, and maintenance for HSR.

## Figures and Tables

**Table 1:** Parameters of longitudinal profiles.

Groups	*R* (m) and Δ*i* (‰)
A	*R*: 25,000 Δ*i*: 8, 10, …, 30
B	*R*: 10,000, 11,000, …, 30,000 Δ*i*: 10
C	*R*: 10,000, 11,000, …, 30,000 Δ*i*: 20
D	*R*: 10,000, 11,000, …, 30,000 Δ*i*: 30

## Data Availability

The original contributions presented in this perspective study are included in the article. Further inquiries can be directed to the corresponding author.
